# Isorhynchophylline Exerts Antinociceptive Effects on Behavioral Hyperalgesia and Allodynia in a Mouse Model of Neuropathic Pain: Evidence of a 5-HT_1A_ Receptor-Mediated Mechanism

**DOI:** 10.3389/fphar.2020.00318

**Published:** 2020-03-18

**Authors:** Ke-Xin Gao, Qing Zhao, Gang-Ren Wang, Lu Yu, Jia-Yi Wu, Xin Zhao

**Affiliations:** ^1^Department of Pharmacology, School of Medical Science, Ningbo University, Ningbo, China; ^2^Department of Neurology, Putuo Hospital, Shanghai University of Traditional Chinese Medicine, Shanghai, China; ^3^Department of Orthopedics, Shanghai Central Hospital of Chinese People’s Armed Police Force, Shanghai, China; ^4^Department of Traditional Chinese Medicine, Putuo Hospital, Shanghai University of Traditional Chinese Medicine, Shanghai, China

**Keywords:** isorhynchophylline, antinociceptive effect, neuropathic pain, serotonin, 5-HT_1A_ receptor

## Abstract

Chronic neuropathic pain poses a significant health problem, for which effective therapy is lacking. The current work aimed to investigate the potential antinociceptive efficacy of isorhynchophylline, an oxindole alkaloid, against neuropathic pain and elucidate mechanisms. Male C57BL/6J mice were subjected to chronic constriction injury (CCI) by loose ligation of their sciatic nerves. Following CCI surgery, the neuropathic mice developed pain-like behaviors, as shown by thermal hyperalgesia in the Hargreaves test and tactile allodynia in the von Frey test. Repetitive treatment of CCI mice with isorhynchophylline (p.o., twice per day for two weeks) ameliorated behavioral hyperalgesia and allodynia in a dose-dependent fashion (5, 15, and 45 mg/kg). The isorhynchophylline-triggered antinociception seems serotonergically dependent, since its antinociceptive actions on neuropathic hyperalgesia and allodynia were totally abolished by chemical depletion of spinal serotonin by PCPA, whereas potentiated by 5-HTP (a precursor of 5-HT). Consistently, isorhynchophylline-treated neuropathic mice showed escalated levels of spinal monoamines especially 5-HT, with depressed monoamine oxidase activity. Moreover, the isorhynchophylline-evoked antinociception was preferentially counteracted by co-administration of 5-HT_1A_ receptor antagonist WAY-100635. *In vitro*, isorhynchophylline (0.1-10 nM) increased the *Emax* (stimulation of [^35^S] GTPγS binding) of 8-OH-DPAT, a 5-HT_1A_ agonist. Of notable benefit, isorhynchophylline was able to correct co-morbidly behavioral symptoms of depression and anxiety evoked by neuropathic pain. Collectively, these findings confirm, for the first time, the disease-modifying efficacy of isorhynchophylline on neuropathic hypersensitivity, and this effect is dependent on spinal serotonergic system and 5-HT_1A_ receptors.

## Introduction

Neuropathic pain is a devastating public health problem that afflicts around 7% of population worldwide (Bouhassira et al., 2008). It is a kind of chronic pain arising from a lesion or diseases of the somatosensory system, and is characterized by behavioral allodynia and hyperalgesia, as well as spontaneous pain (Bridges et al., 2001; Costigan et al., 2009). This disease poses a huge clinical burden because it is refractory to canonical analgesic drugs, e.g., non-steroidal anti-inflammatory drugs and opioids (Eisenberg et al., 2005; Vo et al., 2009). Furthermore, a poor understanding of its etiology and pathogenesis impedes the development of novel therapeutic agents (Bridges et al., 2001). Currently, recommendatory pharmacological treatment relies on drugs initially delved to fight other neuropsychological disorders such as antidepressants and anticonvulsants (Attal et al., 2010). Although the two categories of drugs have been ranked as first-line treatment against neuropathic pain, some inevitable disadvantages (such as modest efficacy, adverse effects, and low patient compliance) limit their usefulness (Arnér and Meyerson, 1988; Finnerup et al., 2005). Therefore, there is a need to develop novel and more efficacious pharmacological agents against neuropathic pain, and compounds derived from herbals or folks may provide an important option.

**Figure 1:**
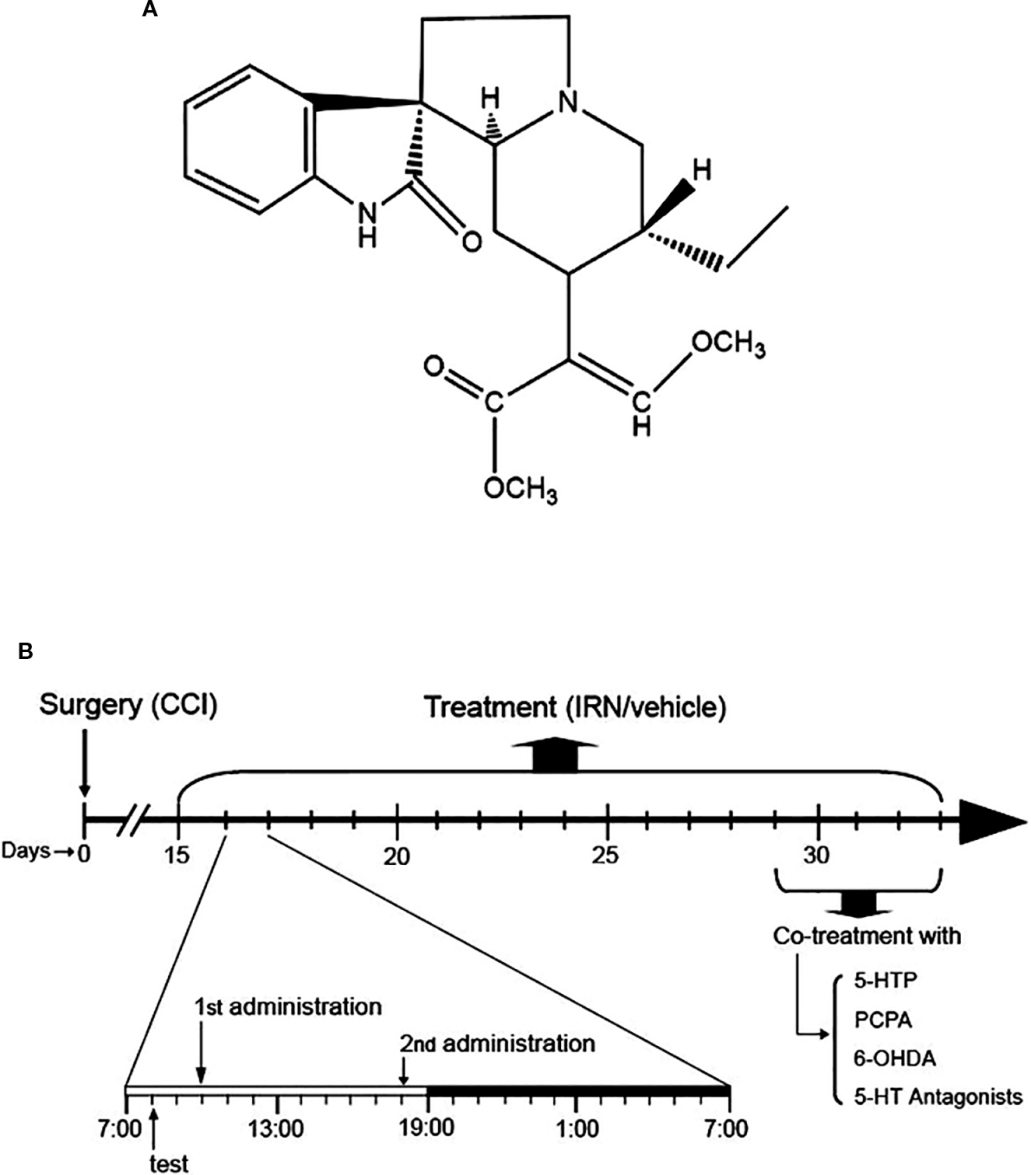
Chemical structure of isorhynchophylline (IRN) and schematic of isorhynchophylline administration. **(A)** Chemical structure of isorhynchophylline. **(B)** Schematic of isorhynchophylline administration. Fifteen days after the surgery of chronic construction injury (CCI), mice were repetitively treated with isorhynchophylline (5, 15, and 45 mg/kg, p.o., twice per day, the first at 10:00 a.m. and the second at 6:00 p.m.). During repetitive isorhynchophylline administration, pain-related behavioral tests were performed 2 h before the morning isorhynchophylline dosing. Following two weeks of isorhynchophylline treatment, PCPA, 6-OHDA, 5-HTP, or 5-HT antagonists were co-administered with isorhynchophylline.

*Uncaria rhynchophylla* (Gou-teng) has been widely used as folk medicine in China for thousands of years, especially for the treatment of cardiovascular and brain disorders (Shi et al., 2003). As an alkaloid compound from *Uncaria rhynchophylla*, isorhynchophylline (IRN, chemical structure shown in **Figure 1A**) has been shown to possess a variety of biological and pharmacological activities, such as anti-tumor, anti-inflammatory, anti-oxidant, anti-diabetic, neuroprotective, and cardiac protective actions (Gan et al., 2011; Xian et al., 2012; Guo et al., 2017; Lee et al., 2017; Zhou et al., 2019), implicating its disease-modifying and health-promoting properties. However, whether isorhynchophylline is able to exert antinociceptive effect remains largely unknown. Recently, it was reported that isorhynchophylline possesses antidepressant-like property in experimental animals (Xian et al., 2017). As antidepressant agents are clinically workable to combat chronic neuropathic pain, we reasoned that isorhynchophylline may also have therapeutic efficacy against neuropathic pain.

In this study, the primary aim was to investigate the antinociceptive effects of isorhynchophylline in a mouse neuropathic pain model, which was produced by loose ligation of the sciatic nerves (CCI model). Furthermore, the mechanisms underlying isorhynchophylline analgesia were explored with a focus on the monoaminergic system, since it is not only extensively involved in the descending pain modulation (Millan, 2002), but also implicated in the antidepressant-like effect of isorhynchophylline (Xian et al., 2017). Finally, as emotional aspects are an intrinsic component of chronic pain, we also evaluated whether isorhynchophylline is able to exert beneficial effects on the co-morbidly anxiogenic-/depressive-like behaviors evoked by chronic neuropathic pain.

## Materials and Methods

### Animals

Adult male C57BL/6J mice (6–7 weeks old upon arrival), obtained from Animal Center of Chinese Academy of Sciences, were used in this study. The animals were housed in group (5–6 per cage) with water and chow available *ad libitum*, 12 h light cycles (on at 07:00 AM), and stable temperature (22 ± 0.5 °C) and relative humidity (60 ± 2%). In animal behavioral tests, operators were blind regarding the treatment cohort and animals were assigned to a cohort randomly. All animal handing and experiments were in accordance with the European Communities Council Directive of 24 November 1986 (86/609/EEC). The animal study was reviewed and approved by the Ningbo University Committee on Animal Care and Use. The authors further ensure here that all efforts were made to minimize animal suffering and the number used.

### Chronic Constriction Injury (CCI) Procedure

A chronic constriction injury (CCI) of ipsilateral sciatic nerve was carried out in mice (7–8 weeks old at the time of surgery shown as day 0) as originally proposed by Bennett and Xie (1988) with minor modifications by us for adaptation to mice (Zhao et al., 2012). Briefly, surgery was performed following the induction of anesthesia by intraperitoneal (i.p.) injection of pentobarbital sodium (60 mg/kg). The right common sciatic nerve was exposed at the nerve of the mid thigh, and close to the sciatic nerve trifurcation, three ligations (4/0 silk threads, with 1 mm spacing appropriately) were tied loosely around it until a slight twitch was seen in the respective hindlimb. The surgical area was dusted with streptomycin and the incision was sutured. For sham animals, the sciatic nerves were isolated without ligations. The animals were placed under a heat lamp until they were awake. CCI or sham-operated mice were housed in separate cages and tested simultaneously. All surgical procedures were performed by the same operator.

### Drugs and Pharmacological Treatment

The administration of isorhynchophylline (p.o., *via* gavage with a volume of 10 ml/kg) was initiated 15 days after the surgery of CCI or sham operation. At this time point, the CCI animals exhibited remarkable nocifensive hypersensitivity, evidenced by thermal hyperalgesia and tactile allodynia. For repetitive dosing, the sham and CCI animals were administered with isorhynchophylline or vehicle twice per day (10:00 AM and 6:00 PM, shown in **Figure 1B**) for two weeks (i.e., from day-15 to day-29 after surgery). After two weeks of isorhynchophylline regimen, the animals were co-administered with 5-hydroxytryptophan (5-HTP, a precursor of serotonin) or one of the 5-HT receptor antagonists (**Figure 1B**). These antagonists are WAY-100635 (a 5-HT_1A_ preferring antagonist), isamoltane (a 5-HT_1B_ preferring antagonist), ritanserin (a 5-HT_2A/2C_ preferring antagonist), and ondansetron (a 5-HT_3_ preferring antagonist). The doses of these agents were used on the basis of previous studies (Wang et al., 2008; Zhao et al., 2012; Hu et al., 2017), with minor modifications to verify paucity of intrinsic activity *per se* in the present nocifensive tests. For i.p. injection, these agents were administered to animals 30 min before isorhynchophylline dosing with a volume of 10 mg/kg. Isorhynchophylline was purchased from Chengdu Mansite Pharmaceutical Co., Ltd. (Chengdu, China) with the purity more than 98%, and the other drugs were from Sigma-Aldrich. Isorhynchophylline was suspended in 0.5% sodium carboxymethyl cellulose and all the other drugs were dissolved in freshly sterile saline.

### Intracerebroventricular, Intrathecal, and Intraplantar Injection

To localize the serotonergic receptors possibly involved, intracerebroventricular (i.c.v.), intrathecal (i.t.) or intraplantar (i.pl.) injection of WAY-100635 was carried out after two weeks of isorhynchophylline regimen. We did two i.c.v., i.t. or i.pl. injections on day-29, 30 min before the morning and the evening isorhynchophylline dosing. The mice were then challenged for nociceptive tests on the next morning, i.e., 38 h after the last isorhynchophylline dosing alone. These procedures are based on previous reports (Choucair-Jaafar et al., 2009; Yalcin et al., 2009; Zhao et al., 2012) regarding the analgesic mechanism of antidepressants.

Intracerebroventricular injections were performed as delineated by Haley and Mccormick (1957). Briefly, under ether anesthesia, mice were grasped firmly with the soft skin behind the head. A 29-gauge needle connecting a Hamilton syringe (50 μl) was inserted perpendicularly into mouse lateral ventricle, through the skull and around 2.0 mm into brain. The injection site was appropriate 1 mm lateral to the midpoint on a line between two anterior bases of the ears. After behavioral tests, the animals were decapitated rapidly and brains were removed and frozen in −70°C. The brains were sectioned in the coronal plane with the thickness of 10μm. The sections were then stained by cresyl violet to verify the injection sites. The successful injection rate was normally more than 95% in our group.

The procedures for intrathecal injections were based on the method described by Hylden and Wilcox (1980). Shortly, a 29-gauge needle a Hamilton syringe (50 μl) was inserted into the sub-arachnoidal space between L5 and L6 vertebrate. The successful placement of the needle was verified by the sign of a tail flick movement. The volume for i.t. injection was 10μl.

The intraplantar injections were performed with ether anesthesia. A 29-gauge injection needle punctured the ipsilateral plantar skin and reached the subcutaneous space proximal to the footpad. The volume for i.pl. injection was 10μl.

### Chemical Ablation of Descending Serotonin (5-HT) and Noradrenaline (NA)

Following two weeks of isorhynchophylline treatment, pharmacological manipulations were carried out to ablate spinal 5-HT or NA in mice. For chemically ablating spinal NA transmission, the mice were intrathecally injected with 6-OHDA (a catecholaminergic neurotoxin) at the dose of 20 μg per mouse (Suzuki et al., 2008; Zhao et al., 2012). To deplete descending 5-HT, *p*-chlorophenylalanine (PCPA, an inhibitor of 5-HT synthesis) was administered i.p. to mice for five successive days at the dose of 300 mg/kg/day (Tanabe et al., 2007; Zhao et al., 2012). 6-OHDA was dissolved in 5 μl of 0.9% saline containing ascorbic acid (100 μg/ml) and PCPA was suspended in 0.5% gum acacia/physiological saline.

### Behavioral Tests

#### Behavioral Tests Overview

Nocifensive behaviors of mice were evaluated by Hargreaves test and von Frey test, with thermal (heat) hyperalgesia and tactile allodynia being used as outcome measurements. The anxiogenic-like behaviors in mice were assayed by light-dark test (LDT) and novelty suppressed feeding test (NSFT), and depressive-like behaviors were measured by tail suspension test (TST) and NSFT. In the tests of LDT, NSFT, and TST, each animal was challenged only once, while in the Hargreaves test and von Frey test, the animals were tested repetitively in a time-course manner for the cohorts of repetitive isorhynchophylline or vehicle treatment. To measure the acute effects of isorhynchophylline, behavioral tests were carried out at different time points (e.g., 0.5, 1, 2, and 4 h) following a single isorhynchophylline administration. In the setting of repetitive isorhynchophylline regimen, the behavioral tests were carried out 2 h before the morning isorhynchophylline administration (shown in **Figure 1B**) as adopted by previous studies (Yalcin et al., 2009; Zhao et al., 2012).

#### Hargreaves Test

Thermal hyperalgesia to heat stimuli was evaluated based on the Hargreaves procedure (Hargreaves et al., 1988), with slight modification for adaptation to mice, using a Plantar test apparatus (Ugo Basile, Italy). Mice were placed in clear Plexiglas cubicles for acclimatization at least 20 min. A constant intensity radiant heat source (approximate 10 s for naïve mice) was focused on the paw of mice and the thermal (paw withdraw) latency was defined as the time (seconds) from the initiation of heat exposure to the hind paw withdrawal. A cut-off time was set at 25 s to avoid tissue damage.

#### Von Frey Test

Sensory thresholds for allodynia to tactile stimuli were assayed using a series of von Frey filaments (Stoelting, USA) as described previously (Yalcin et al., 2009; Zhao et al., 2012). Shortly, animals were placed in transparent Plexiglas cubicles equipped with an elevated wire mesh floor and allowed to a 20-min acclimation before testing. The filaments in an ascending order were applied perpendicularly to the plantar surface of hind paws. Each filament was tested five times per paw, and a positive response was defined as an immediate and robust withdrawal of the hind paw. The tactile threshold was set as the minimal force (gram) that leaded to at least three withdrawals out of five successive trials, with a cut-off force of 10 g.

#### Light-Dark Test (LDT)

The apparatus consists of light and dark boxes (25 × 18 × 20 cm each) connected by a dark tunnel (9 × 7 × 6 cm). The lit compartment was brightly illuminated (1,500 lux). Mice were placed in the dark compartment in the beginning of the test and the time spent in the lit compartment was recorded during 5 min (Zhao et al., 2018).

#### Novelty Suppressed Feeding Test (NSFT)

The testing apparatus consists of a 40×40×30 cm plastic box with floor covered with 1 cm sawdust. Fast mice were placed in a corner of a square arena where a single pellet of food was placed on a white paper positioned in the box center. The latency to first contact or chew the pellet was recorded within a 5-min period. This procedure elicits a conflict between the drive to eat the food and the fear of venturing in the center of the arena (Zhao et al., 2018).

#### Tail Suspension Test (SPT)

The procedure was established initially by Steru et al. (1985), with a minor revision by our group (Hu et al., 2017). Shortly, mice were suspended (50 cm above the floor) for a 6 min testing session with an adhesive tape affixed 1 cm from the tip of the tail. Immobility was defined as the complete cessation of movement while suspended. The immobility time was recorded during the entire 6 min testing session.

### Measurement of Monoamines (Metabolites) and Monoamine Oxidase (MAO) Activity

After 3 weeks isorhynchophylline or vehicle treatment, the sham or CCI mice were decapitated, followed by the lumbar enlargements (approximately 1 cm) of their spinal cords being removed and tissues being dissected rapidly on an ice-chilled glass plate. The tissue samples were weighed, and then stored at −80°C. The contents of monoamines (NA, serotonin and dopamine) and their metabolites (5-HIAA and DOPAC) were measured as described previously by high-performance liquid chromatography (HPLC) with electrochemical detection (Zhao et al., 2014). Briefly, each spinal cord was homogenized by ultrasonication in 200 μl of 0.4 M perchloric acid (solution A). The homogenate was kept on ice for 1 h and then centrifuged at 12,000 × g at 4°C for 20 min. After discarding the pellet, an aliquot of 160 μl of supernatant was added to 80 μl of solution B (containing 0.2 M potassium citrate, 0.3 M dipotassium hydrogen phosphate and 0.2 M EDTA). The mixture was kept on ice for 1 h and then centrifuged at 12, 000 × g at 4°C for 20 min again. 20 μl of the supernatant was directly injected into an ESA liquid chromatography system equipped with a reversed-phase C_18_ column (150 × 4.6 mm I.D., 5 μm) and an electrochemical detector (ESA CoulArray, Chelmstord, MA, USA.). The mobile phase consisted of 125 mM citric acid –sodium citrate (PH 4.3), 0.1 mM EDTA, 1.2 mM sodium octanesulfonate, and 16% methanol. The flow rate was 1.0 ml/min. the tissue levels of monoamines and their metabolites were expressed as nanograms per gram wet spinal tissue. The MAO activity in the tissue was assayed according to our established protocol (Zhao et al., 2014).

### *In Vitro* Procedures

#### Cell Culture

Chinese Hamater Ovary (CHO) cells expressing human 5-HT_1A_ receptors (a generous gift from Dr Yu Yang) were cultured at 37°C and 5% CO_2_ in Ham’s F-12 medium fortified with 10% FBS, 200 μg/ml geneticin, and 600 mg/ml G418.

#### Cell Culture Receptor Preparation

Cells were harvested by trypsinization and centrifuged at low speed in ice-cold medium. The pellet was re-suspended in ice-cold Earle’s Balanced Salt Solution followed by centrifugation. Cells were re-suspended in 10 ml of ice-cold binding buffer (10 μM pargyline, 4 mM CaCl2, 50 mM Tris, pH 7.4) homogenized with Teflon-glass, and centrifuged for 45,000 g-min at 4°C. To yield a crude membrane preparation, the pellet was re-suspended in 30 ml of ice-cold binding buffer, and homogenized first with Teflon-glass and then with a Polytron for 5 s. The receptor preparation was stored on ice and assayed within the next 1 h.

#### Radioligand Displacement Assay

Each assay was performed with 0.7 nM [^3^H]-8-OH-DPAT and human 5-HT_1A_ CHO cell membranes (50 μg per well) using Tris-binding buffer (0.1% BSA, 50 mM Tris-base, 50 mM Tris-HCl, pH 7.4) in a total volume of 500 μl. All assays were carried out at 37°C for 60 min followed by addition of ice-cold Tris-binding buffer and vacuum filtration. Specific binding was defined by the presence and absence of 1 μM unlabelled 8-OH-DPAT.

#### [35S]-GTPγS Binding Assay

Each assay was done with human 5-HT_1A_ CHO cell membranes (50 μg protein per well), 0.1 nM [35S]-GTPγS, GTPγS binding buffer (50 mM Tris-HCl; 50 mM Tris-Base; 5 mM MgCl_2_; 1 mM EDTA; 100 mM NaCl; 1 mM dithiothreitol; and 0.1% BSA), and 30 μM GDP, in a final volume of 500 μl. Non-specific binding was measured in the presence of 30 μM GTPγS. Assays were performed at 30°C for 60 min.

### Statistical Analysis

All values are presented as the mean ± S.E.M. Data were analyzed by multifactor analysis of variance (ANOVA) or one-way ANOVA. For multifactor-ANOVA, for example, the surgery (sham or CCI) and the treatment (vehicle or drug administration) were done as between-group factors. When needed, the time of measurement (time-course data) was done as within-subject factor. The Tukey test was used for *post hoc* comparisons. For one-way ANOVA, Student-Newman-Keuls test was used for multiple comparisons, followed by Student’s test to evaluate the difference between two groups at the same time. Differences with *p* < 0.05 were considered statistically significant.

## Results

### Chronic Isorhynchophylline Treatment Ameliorated Thermal (Heat) Hyperalgesia and Tactile Allodynia in Neuropathic Mice

**Figure 2:**
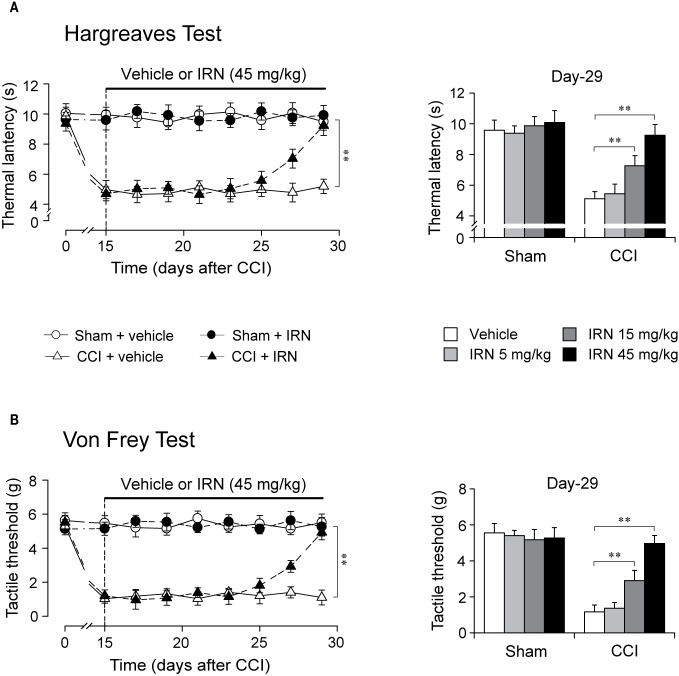
Effects of repetitive isorhynchophylline (IRN) treatment on thermal (heat) and tactile hypersensitivity in neuropathic (CCI) mice. **(A)** Repetitive isorhynchophylline treatment (45 mg/kg, p.o., twice per day for 14 days) corrected thermal (heat) hyperalgesia in neuropathic mice (time-course measures shown in left panel) and this antihyperalgesia was dose-dependent (5, 15, and 45 mg/kg, right panel showing values assayed on day 29), without affecting the profiles in sham-operated mice. **(B)** Repetitive isorhynchophylline treatment (45 mg/kg, p.o., twice per day for 14 days) corrected tactile allodynia in neuropathic mice (time-course measures shown in left panel) and this antiallodynia was dose-dependent (5, 15, and 45 mg/kg, right panel showing values assayed on day 29), without affecting the profiles in sham-operated mice. Data are expressed as mean ± SEM (n = 9–11 per group), evaluated by multifactor ANOVA followed by Duncan test and one-way ANOVA followed by Student-Newman-Keuls test. **p < 0.01, significantly different from the vehicle group.

The mononeuropathy produced by chronic constriction injury of the sciatic nerve evoked in mice ipsolateral thermal hyperalgesia and tactile allodynia, which were persistent and maintained throughout the experimental period (**Figures 2A, B**, left panels), without affecting the measurements of contra-lateral paws (data not shown). As expected, sham-surgery operation did not change the nocifensive reponsivity to tactile or thermal stimuli.

After two weeks of treatment as shown in **Figure 2**, isorhynchophylline strikingly attenuated in neuropathic mice the evoked thermal hyperalgesia (surgery × treatment × days interaction, F_7,282_ = 9.8, *p* = 0.0023; *post hoc*: CCI + vehicle = CCI + IRN < sham on days 15–25, CCI + vehicle < all other groups on days 27–29; **Figure 2A**, left panel) and tactile allodynia (surgery × treatment × days interaction, F_7,282_ = 13.1, *p* = 0.0008; *post hoc*: CCI + vehicle = CCI + IRN < sham on days 15–25, CCI + vehicle < all other groups on days 27-29; **Figure 2B**, left panel) in a dose-related manner (**Figures 2A, B**, right panels), showing statistical difference at doses of 15 mg/kg and 45 mg/kg. At the high dose of 45 mg/kg, isorhynchophylline showed recuperating effects on the evoked nociceptive hypersensitivities (heat hyperalgesia and tactile allodynia), without influencing the measures in sham-operated mice (**Figures 2A, B**). In the experimental session of acute treatment, isorhynchophylline did not show any favorable effect on these nocifensive measurements in sham-operated and neuropathic animals (Supplementary Material files **Figure S1**).

To determine whether isorhynchophylline-induced behavioral alterations in the Heagreaves test and von Frey test have relevance to the effect of isorhynchophylline on motor function, we performed another set of experiments to probe the effects of repetitive isorhynchophylline treatment on motor ability and locomotor activity in neuropathic mice. Our results show repetitive isorhynchophylline treatment, at the dose range of 5–45 mg/kg, did not affect the behavioral outcomes in the locomotor test (Supplementary Material files **Figure S2A**) and rota-rod test (Supplementary Material files **Figure S2B**). These results indicate isorhynchophylline-induced behavioral alterations in pain-related assays should not be interfered by potential effect on motor function.

### Interruption and Re-Initiation of Treatment

**Figure 3:**
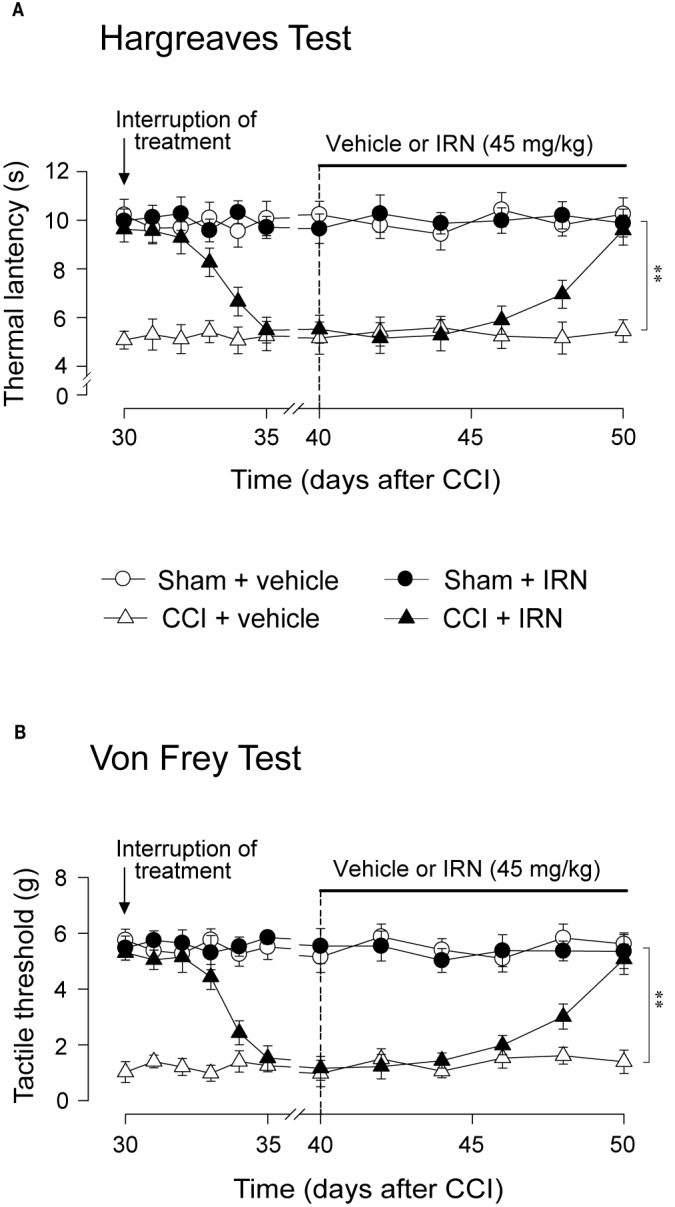
Effect of treatment cessation on the therapeutic actions of isorhynchophylline (IRN) upon neuropathic hypersensitivity in neuropathic mice. Following treatment cessation, the analgesic actions of isorhynchophylline (45 mg/kg, p.o., twice per day) on thermal hyperalgesia **(A)** and tactile allodynia **(B)** lasted for three days. Data are expressed as mean ± SEM (n = 9–11 per group), assessed by multifactor ANOVA followed by Duncan test. **p < 0.01, significantly different from the vehicle group.

As isorhynchophylline at the high dose of 45 mg/kg exerted disease-modifying effects on neuropathic hyperalgesia and allodynia, we evaluated how long can these actions last, and whether these actions can be re-initiated following isorhynchophylline cessation. As shown in **Figure 3**, we terminated isorhynchophylline dosing on day-30 when the hypernociceptive behaviors (heat hyperalgesia and tactile allodynia) were normalized by repetitive isorhynchophylline treatment. Although the interruption of isorhynchophylline dosing caused the relapse of neuropathic hyperalgesia (**Figure 3A**) and allodynia (**Figure 3B**), its therapeutic actions lasted for at least 3 days. On day-40, a new repetitive dosing of isorhynchophylline (45 mg/kg, p.o., twice per day) was initiated. In this session, isorhynchophylline again produced disease-modifying actions on neuropathic hyperalgesia (**Figure 3A**) and allodynia (**Figure 3B**) with remarkably less time of onset (around 10 days) compared with the first session of regimen.

### Repetitive Isorhynchophylline Treatment Increased Spinal Monoamines Levels and Reduced Monoamine Oxidase (MAO) Activity in Neuropathic Mice

**Figure 4:**
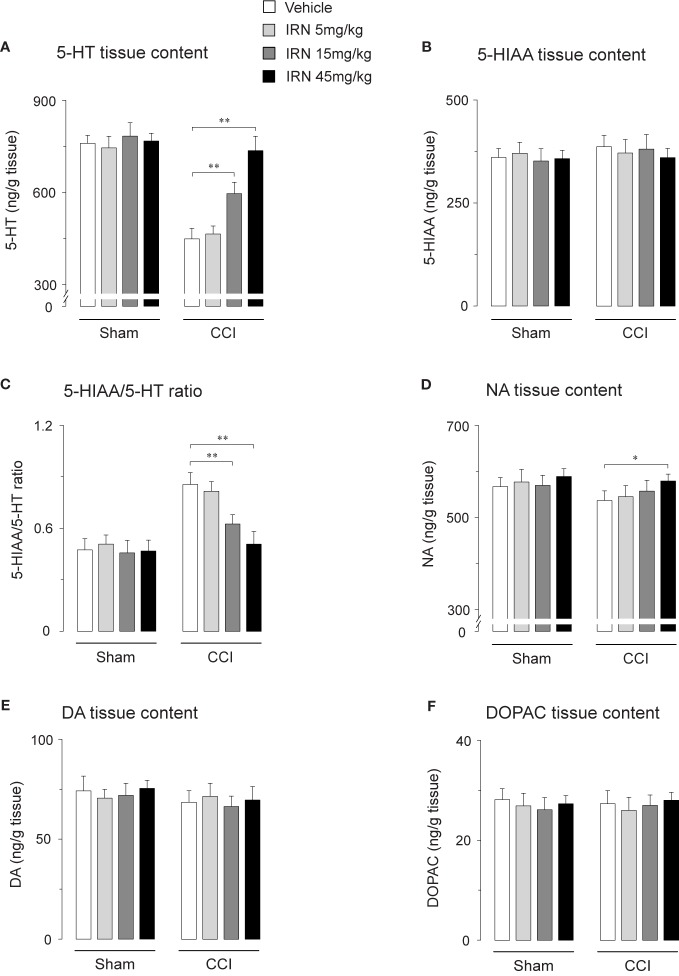
Effect of repetitive isorhynchophylline (IRN) treatment on the tissue contents of monoamines and their metabolites in the spinal cord. **(A)** Repetitive isorhynchophylline treatment dose-dependently increased the spinal level of 5-HT in CCI mice, without influencing the measures in sham-operated mice. **(B)** Repetitive isorhynchophylline treatment did not alter the spinal level of 5-HIAA in sham-operated and CCI mice. **(C)** Repetitive isorhynchophylline treatment dose-dependently reduced spinal 5-HIAA/5-HT ratio in CCI but not sham-operated mice. **(D)** Repetitive isorhynchophylline treatment slightly increased, with a marginal difference at the high dose of 45 mg/kg, the spinal level of NA in CCI mice, without influencing the measures in sham-operated mice. **(E)** Repetitive isorhynchophylline treatment did not alter the spinal level of DA in sham-operated and CCI mice. **(F)** Repetitive isorhynchophylline treatment did not alter the spinal level of DOPAC in sham-operated and CCI mice. Data are expressed as mean ± SEM (n = 9-11 per group), assessed by one-way ANOVA followed by Student-Newman-Keuls test. ^*^p < 0.05 and ^**^p < 0.01, significantly different from the vehicle group.

As monoaminergic system, a vital ingredient of descending pathway in pain regulation (Millan, 2002), is involved in the antidepressant-like effects of isorhynchophylline (Xian et al., 2017), we reasoned that modification of descending monoamine transmission by isorhynchophylline may contribute its antinociceptive effects. We therefore investigated the effect of repetitive isorhynchophylline treatment on spinal levels of monoamines. Compared with vehicle treatment, isorhynchophylline dose-dependently increased, in neuropathic mice, the spinal level of 5-HT (F_3,38_ = 17.3, *p* = 0.0026; **Figure 4A**), and decreased the ratio of 5-HIAA/5-HT (F_3,38_ = 8.4, *p* = 0.0076; **Figure 4C**). However, isorhynchophylline at the dose range of 5–45 mg/kg, did not alter the levels of spinal 5-HT and the ratio of 5-HIAA/5-HT in sham-operated mice. Regarding spinal NA level (**Figure 4D**), there is a marginal but significant increase (F_3,38_ = 12.7, *p* = 0.0376) in neuropathic mice following treatment with isorhynchophylline at high dose (45 mg/kg). The present isorhynchophylline regimen did not change the spinal levels of 5-HIAA (**Figure 4B**), dopamine (**Figure 4E**) and DOPAC (**Figure 4F**) in sham-operated and neuropathic mice. Consistent with previous studies (Vogel et al., 2003), there is a sharp serotonin-fall in vehicle-treated neuropathic mice, indicating the dearth of serotonin at spinal level may have relevance on peripheral nerve injury-induced mononeuropathy.

Escalated spinal 5-HT level and depressed ratio of 5-HIAA/5-HT imply the alteration of monoamine metabolism by isorhynchophylline. The monoamine in CNS is metabolized by two isoforms of MAO, i.e., MAO-A and MAO-B. We then evaluated the spinal MAO-A and MAO-B activities following repetitively oral isorhynchophylline treatment. As shown in **Table 1**, Neuropathic mice showed escalated activity of MAO-A but not MAO-B compared with sham mice, which is consistent with a previous study by Villarinho et al. (2013). Following repetitive oral treatment, isorhynchophylline with doses ranging from 5 to 45 mg/kg dose-dependently inhibited the escalated spinal MAO-A activity in neuropathic mice (F_3,38_ = 14.2, *p* = 0.0072), and at the high dose of 45 mg/kg the MAO-A activity is reduced to the level essentially equivalent to that of sham mice. The present isorhynchophylline regimen did not influence the spinal MAO-B activity in both sham and neuropathic mice.

**Table 1:** Effect of isorhynchophylline (IRN) on spinal type A and type B monoamine oxidase activities in mice.

Group	Monoamine oxidase-A activity (nmol/30 min/mg protein)		Monoamine oxidase-B activity (nmol/30 min/mg protein)	
	Sham mice	CCI mice	Sham mice	CCI mice
Vehicle	118.5 ± 6.7	168.7 ± 8.5^##^	146.3 ± 8.7	150.6 ± 7.2
IRN-5 mg/kg	114.0 ± 8.1	172.6 ± 8.9	143.1 ± 7.4	152.3 ± 8.7
IRN -15 mg/kg	125.2 ± 7.0	143.8 ± 7.6^**^	151.4 ± 9.6	144.9 ± 8.9
IRN -45 mg/kg	121.2 ± 7.8	129.8 ± 9.8^**^	141.4 ± 8.5	147.8 ± 9.7

Values were expressed as mean values ± SEM of 9-10 mice.

^##^p < 0.01, compared with vehicle treated Sham mice.

**p < 0.01, compared with vehicle treated CCI mice.

### The Antinociceptive Effects of Isorhynchophylline Were Abolished by Chemical Ablation of Spinal 5-HT, While Potentiated By Co-Treatment With 5-HTP

**Figure 5:**
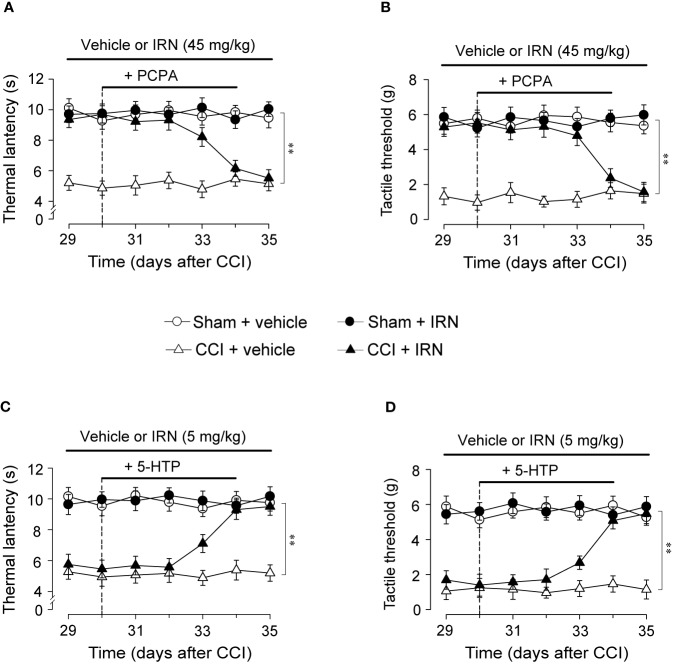
Effects of co-administration of PCPA and 5-HTP on the antinociceptive effects of isorhynchophylline (IRN) on thermal (heat) hyperalgesia and tactile allodynia. **(A)** Depletion of serotonin by PCPA (300 mg/kg for 5 days, i.p.) abrogated the antinociceptive action of isorhynchophylline on thermal (heat) hyperalgesia in CCI mice, without influencing the thermal (heat) sensitivity in sham-operated and vehicle-treated CCI mice. **(B)** Depletion of serotonin by PCPA (300 mg/kg for 5 days, i.p.) abrogated the antinociceptive action of isorhynchophylline on tactile allodynia in CCI mice, without influencing the tactile sensitivity in sham-operated and vehicle-treated CCI mice. **(C)** Administration of 5-HTP (10 mg/kg, i.p.) or isorhynchophylline at ineffective dose (5 mg/kg, p.o.) alone did not exhibit any antihyperalgesic effect in the Hargreveas test regardless of sham or CCI mice, but co-administration of them produced marked antinociceptive effect on thermal (heat) hyperalgesia in CCI (not sham-operated) mice. **(D)** Administration of 5-HTP (10 mg/kg, i.p.) or isorhynchophylline at ineffective dose (5 mg/kg, p.o.) alone did not exhibit any antiallodynic effect in the von Frey test regardless of sham or CCI mice, but co-administration of them engendered remarkable antinociceptive effect on tactile allodynia in CCI (not sham-operated) mice. Data are expressed as mean ± SEM (n = 9–11 per group), assessed by multifactor ANOVA followed by Duncan test. **p < 0.01, significantly different from the vehicle group.

To explore whether the monoamine system is causally responsible for the antinociceptive effects of isorhynchophylline, we observed the influence of ablating spinal 5-HT or NA on the antinociceptive effects of isorhynchophylline. Consistent with our previous study (Zhao et al., 2014), a single intrathecal injection of 6-OHDA (20 μg per mouse) or five consecutive injection of PCPA (i.p., 300 mg/kg, once per day) reduced spinal NA or 5-HT content, respectively (data not shown). We then evaluated the effect of 5-HT or NA ablation by PCPA or 6-OHDA, respectively, on the antinociceptive effects of isorhynchophylline in neuropathic mice. As shown in **Figure 5**, PCPA (300 mg/kg, i.p.), after 5 days of successive injections, reverted the actions of isorhynchophylline on thermal hyperalgesia (surgery × treatment × days interaction, F_6,232_ = 9.7, *p* = 0.0092; *post hoc*: CCI + vehicle < all other groups on days 29–33, CCI + vehicle = CCI + IRN < sham on days 34–35; **Figure 5A**) and tactile allodynia (surgery × treatment × days interaction, F_6,232_ = 15.9, *p* = 0.0057; *post hoc*: CCI + vehicle < all other groups on days 29-33, CCI + vehicle = CCI + IRN < sham on days 34-35; **Figure 5B**), pointing to a critical role for serotonergic system in the present isorhynchophylline analgesia. In contrast, 6-OHDA-induced spinal NA ablation did not impact on the antinociception exerted by isorhynchophylline (Supplementary Material files **Figure S3**), indicating NA system may not be involved.

To further validate the crucial involvement of the serotonergic system, we tested whether the present isorhynchophylline analgesia can be affected by co-treatment with 5-HTP, a precursor of serotonin. As shown in **Figure 5**, a single administration of 5-HTP (10 mg/kg, i.p.) or ineffective dose of isorhynchophylline (5 mg/kg, p.o.) did not impact on the profiles measured in the Hargreaves test and von Frey test for both sham and neuropathic mice. However, co-administration of them yielded marked antinociceptive effects on thermal hyperalgesia (surgery × treatment × days interaction, F_6,246_ = 6.4, *p* = 0.0067; *post hoc*: CCI + vehicle = CCI + IRN < sham on days 29–32, CCI + vehicle < all other groups on days 33–35; **Figure 5C**) and tactile allodynia (surgery × treatment × days interaction, F_6,246_ = 21.6, *p* = 0.0029; *post hoc*: CCI + vehicle = CCI + IRN < sham on days 29–32, CCI + vehicle < all other groups on days 33–35; **Figure 5D**) in neuropathic mice, pointing toward a potentiating effect when isorhynchophylline was co-treated with 5-HTP. These results support the serotonergic mechanism underlying the present isorhynchophylline analgesia.

### Spinal 5-HT_1A_ Receptors Were Essential for the Effects of Isorhynchophylline on Thermal Hyperalgesia and Tactile Allodynia in Neuropathic Mice

**Figure 6:**
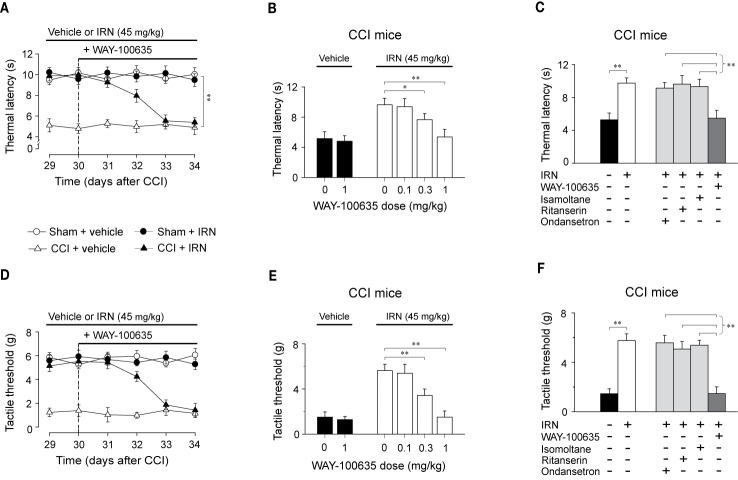
The antinociceptive effects of isorhynchophylline (IRN) on thermal (heat) hyperalgesia and tactile allodynia are mediated by 5-HT_1_ receptors. **(A)** Co-administration of 5-HT_1_ receptor antagonist WAY-100635 (1 mg/kg, i.p.) abrogated the antihyperalgesic effect of isorhynchophylline to thermal (heat) stimuli in CCI mice, without influencing the measures in sham-operated or vehicle-treated CCI mice. **(B)** The antihyperalgesic effect of isorhynchophylline in neuropathic mice, shown as increased thermal latency in Hargreaves test, was counteracted by WAY-100635 in a dose-dependent manner (0.1, 0.3, and 1 mg/kg, i.p.). **(C)** The comparison of the effect of co-administration of WAY-100635, isamoltane, ritanserin, or ondansetron on isorhynchophylline-induced antihyperalgesia in neuropathic mice. **(D)** Co-administration of 5-HT_1_ receptor antagonist WAY-100635 (1 mg/kg, i.p.) abrogated the antiallodynic effect of isorhynchophylline to tactile stimuli in neuropathic mice, without influencing the measures in sham-operated or vehicle-treated neuropathic mice. **(E)** The antiallodynic effect of isorhynchophylline in neuropathic mice, shown as increased tactile threshold in von Frey test, was counteracted by WAY-100635 in a dose-dependent manner (0.1, 0.3, and 1 mg/kg, i.p.). **(F)** The comparison of the effect of co-administration of WAY-100635, isamoltane, ritanserin, or ondansetron on isorhynchophylline-induced antiallodynia in neuropathic mice. Data are expressed as mean ± SEM (n = 9–11 per group), assessed by multifactor ANOVA followed by Duncan test or one-way ANOVA followed by Student-Newman-Keuls test. *p < 0.05 and **p < 0.01, significantly different from the vehicle group.

To disclose the subtype of 5-HT receptors implicated in the antinociceptive effects of isorhynchophylline, we asked whether the isorhynchophylline actions are influenced by subtype-selective 5-HT receptor antagonists, i.e., WAY-100635 (5-HT_1A_ receptor antagonist), isamoltane (5-HT_1B_ receptor antagonist), ritanserin (5-HT_2A/C_ receptor antagonist), and ondansetron (5-HT_3_ receptor antagonist). Our results show that repetitive co-administration of 5-HT_1A_ receptor antagonist WAY-100635 offset the antinociceptive effects of isorhynchophylline on thermal hyperalgesia (surgery × treatment × days interaction, F_5,196_ = 17.1, *p* = 0.0036; *post hoc*: CCI + vehicle < all other groups on days 29–32, CCI + vehicle = CCI + IRN < sham on days 33–34; **Figure 6A**) and tactile allodynia (surgery × treatment × days interaction, F_5,196_ = 5.7, *p* = 0.0047; *post hoc*: CCI + vehicle < all other groups on days 29–32, CCI + vehicle = CCI + IRN < sham on days 33–34; **Figure 6D**). The effects of WAY-100635 on isorhynchophylline-induced antihyperalgesia (F_3,38_ = 6.9, *p* = 0.0081; **Figure 6B**) and antiallodynia (F_3,38_ = 10.2, *p* = 0.0028; **Figure 6E**) are dose-dependent (0.1, 0.3, and 1 mg/kg). In contrast, the other 5-HT antagonists (ondansetron, isamoltane, and ritanserin) did not influence the antinociceptive effects of isorhynchophylline on thermal hyperalgesia (**Figure 6C**) and tactile allodynia (**Figure 6F**), further consolidating the allegation that 5-HT_1_ receptors are exclusively engaged in the present isorhynchophylline analgesia.

**Figure 7:**
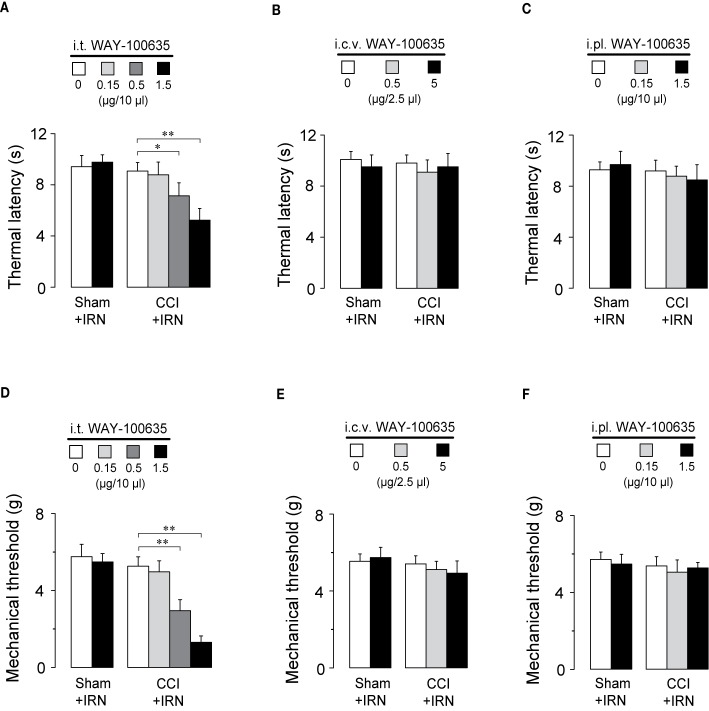
The spinal 5-HT_1_ receptors are responsible for the antinociceptive effects of isorhynchophylline (IRN) on behavioral hyperalgesia and allodynia. **(A)** I.t. injection of WAY-100635 counteracted the antihyperalgesic effect of isorhynchophylline to thermal (heat) stimuli in a dose-dependent manner (0.15, 0.5 and 1.5 μg). **(B)** Effect of intracerebroventricular (i.c.v., 0.5 and 5 μg) injection of WAY-100635 on the thermal latency in sham-operated and neuropathic mice. **(C)** Effect of intraplantar (i.pl., 0.15 and 1.5 μg) injection of WAY-100635 on the thermal latency in sham-operated and neuropathic mice. **(D)** I.t. injection of WAY-100635 counteracted the antiallodynic effect of isorhynchophylline to tactile stimuli in a dose-dependent manner (0.15, 0.5, and 1.5 μg). **(E)** Effect of intracerebroventricular (i.c.v., 0.5 and 5 μg) injection of WAY-100635 on the tactile threshold in sham-operated and neuropathic mice. **(F)** Effect of intraplantar (i.pl., 0.15 and 1.5 μg) injection of WAY-100635 on the tactile threshold in sham-operated and neuropathic mice. Data are expressed as mean ± SEM (n = 8–10 per group), assessed by two-way ANOVA followed by Duncan test. *p < 0.05 and **p < 0.01, significantly different from the vehicle group.

To locate the functional involvement of 5-HT_1_ receptors in the antinociceptive effects of isorhynchophylline, we injected WAY-100635 *via* three special delivery routes (i.t., i.c.v., and i.pl.) and tested its effect on the analgesia by isorhynchophylline. As shown in **Figures 7A, D**, i.t. injection of WAY-100635 counteracted, in a dose-dependent manner, the antinociceptive effects of isorhynchophylline on thermal hyperalgesia (**Figure 7A**, F_3,38_ = 23.4, *p* = 0.0093) and tactile allodynia (**Figure 7D**, F_3,38_ = 7.7, *p* = 0.0056) in neuropathic mice, without influencing the measures in sham mice. However, i.c.v. (5 μg in 2.5 μl, **Figures 7B, E**) or i.pl. (1.5 μg in 10 μl, **Figures 7C, F**) injection of WAY-100635 did not impact on the nociceptive responses to thermal (**Figures 7B, C**) and tactile (**Figures 7E, F**) stimuli in isorhynchophylline-treated sham and neuropathic mice. These results indicate the 5-HT_1_ receptors at spinal site are responsible for the present isorhynchophylline analgesia in neuropathic mice.

### Isorhynchophylline Enhanced the Activation of 5-HT_1A_ Receptor by 8-OH-DPAT, a 5-HT_1A_ Receptor Agonist, in CHO Cell Membranes

**Figure 8:**
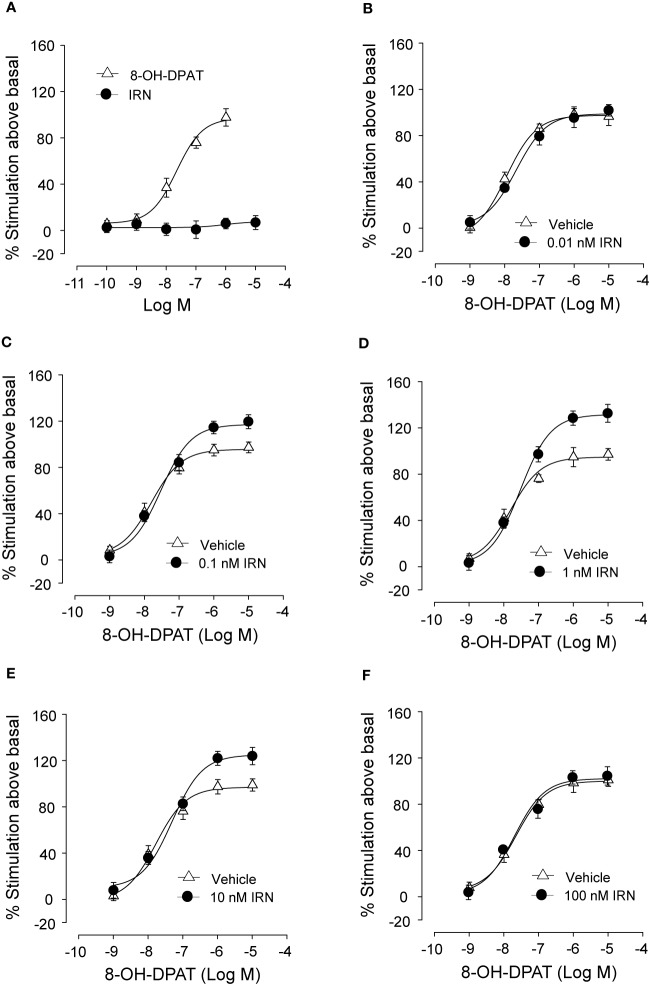
*In vitro* procedures revealing the enhancement of 5-HT_1A_ receptor activation by isorhynchophylline (IRN). **(A)** Effect of 8-OH-DPAT (n = 10) and isorhynchophylline (n = 10) on [^35^S] GTPγS binding to 5-HT_1A_ expressing human CHO cell membranes. **(B)** Effect of 8-OH-DPAT on [^35^S] GTPγS binding to 5-HT_1A_ expressing human CHO cell membranes in the presence of vehicle (n = 10) or 0.01 nM isorhynchophylline (n = 10). **(C)** Effect of 8-OH-DPAT on [^35^S] GTPγS binding to 5-HT_1A_ expressing human CHO cell membranes in the presence of vehicle (n = 10) or 0.1 nM isorhynchophylline (n = 10). **(D)** Effect of 8-OH-DPAT on [^35^S] GTPγS binding to 5-HT_1A_ expressing human CHO cell membranes in the presence of vehicle (n = 9) or 1 nM isorhynchophylline (n = 9). (E) Effect of 8-OH-DPAT on [^35^S] GTPγS binding to 5-HT_1A_ expressing human CHO cell membranes in the presence of vehicle (n = 10) or 10 nM isorhynchophylline (n = 10). **(F)** Effect of 8-OH-DPAT on [^35^S] GTPγS binding to 5-HT_1A_ expressing human CHO cell membranes in the presence of vehicle (n = 9) or 100 nM isorhynchophylline (n = 9). Symbols represent mean values ± SEM.

As shown in **Figure 8A**, isorhynchophylline by itself did not produce any significant stimulation upon [^35^S] GTPγS binding to 5-HT_1A_ CHO cell, with concentrations ranging from 0.1 nM to 10 μM. As expected, the 5-HT_1A_ agonist 8-OH-DPAT produced remarkable stimulant effect on 5-HT_1A_ receptors shown as a significant increase of [^35^S] GTPγS binding to 5-HT_1A_ CHO cell, and this action is concentration-dependent with the range of 1–1,000 nM (**Figure 8A**). Intriguingly, we found that isorhynchophylline, at the concentration range of 1–100 nM, induced a statistically increase in the efficacy (*E_max_*), but not in the potency (*EC_50_*), with which 8-OH-DPAT stimulates [^35^S]-GTPγS binding to 5-HT_1A_ expressing CHO cell membranes (**Figures 8B–F** and **Table 2**). Furthermore, we found that in the same membranes, isorhynchophylline at the concentration range of 1 nM-10 μM did not show any significant activity to displace the binding of [^3^H]-8-OH-DPAT (Supplementary Material files **Figure S4A**). Also within the effective concentration range of 0.1–10 nM for the enhancement of [^35^S] GTPγS binding, isorhynchophylline did not influence the binding of 8-OH-DPAT to 5-HT_1A_ receptors (Supplementary Material files **Figures S4B–D**). Collectively, these *in vitro* data demonstrate that isorhynchophylline can modulate the activation of 5-HT_1A_ indirectly, but not by binding to their orthosteric sites directly.

**Table 2:** Effects of isorhynchophylline (IRN), at the concentration range of 0.01-100 nM, on mean EC_50_ and *E*_max_ values of 8-OH-DPAT for its stimulation of [^35^S] GTPγS binding to 5-HT_1A_ expressing CHO cell membranes.

Pretreatment	Mean EC_50_ (nM)	95% confidence limits (nM)	Mean E_max_ (%)	95% confidence limits (nM)	n
vehicle	15.4	9.7–24.6	98.7	92.4–105.2	10
0.01 nM IRN	18.2	10.5–31.5	101.6	95.3–107.7	10
vehicle	14.3	9.2–22.1	97.8	92.5–103.3	9
0.1 nM IRN	28.7	15.5–53.2	120.3^**^	113.0–127.9	9
vehicle	13.9	7.8–24.8	98.3	90.5–106.4	10
1 nM IRN	26.3	14.5–47.9	132.9^**^	123.4–142.8	10
vehicle	15.8	8.9–27.8	99.7	93.2–106.4	10
10 nM IRN	32.4	17.9–58.8	124.8^**^	117.3–132.2	10
vehicle	15.6	9.6–25.3	101.7	94.5–109.1	9
100 nM IRN	16.3	9.4–28.3	104.8	98.0–110.9	9

**p < 0.01, compared with respective vehicle group (values in the previous row). See also **Figure 8**.

### Chronic Isorhynchophylline Treatment Corrected Co-Morbidly Behavioral Symptoms of Depression and Anxiety in Neuropathic Mice

A separate set of experiment was performed to evaluate whether isorhynchophylline is able to engender favorable effects on co-morbid depressive- and anxiety-like behaviors in neuropathic mice. Congruent with previous studies (Hu et al., 2009; Zhao et al., 2014), neuropathic mice developed co-morbidly depressive-/anxiogenic-like behaviors 2 weeks after the CCI surgery, as evidenced by increased immobility time in the tail suspension test (TST, a measurement of depressive-like behavior), increased latency to feeding in the novelty suppressed feeding test (NSFT, a measurement indicative of depressive- and anxiety-like behavior), and decreased staying time in lit part in the light-dark test (LDT, a measurement of anxiety-like behavior).

**Figure 9:**
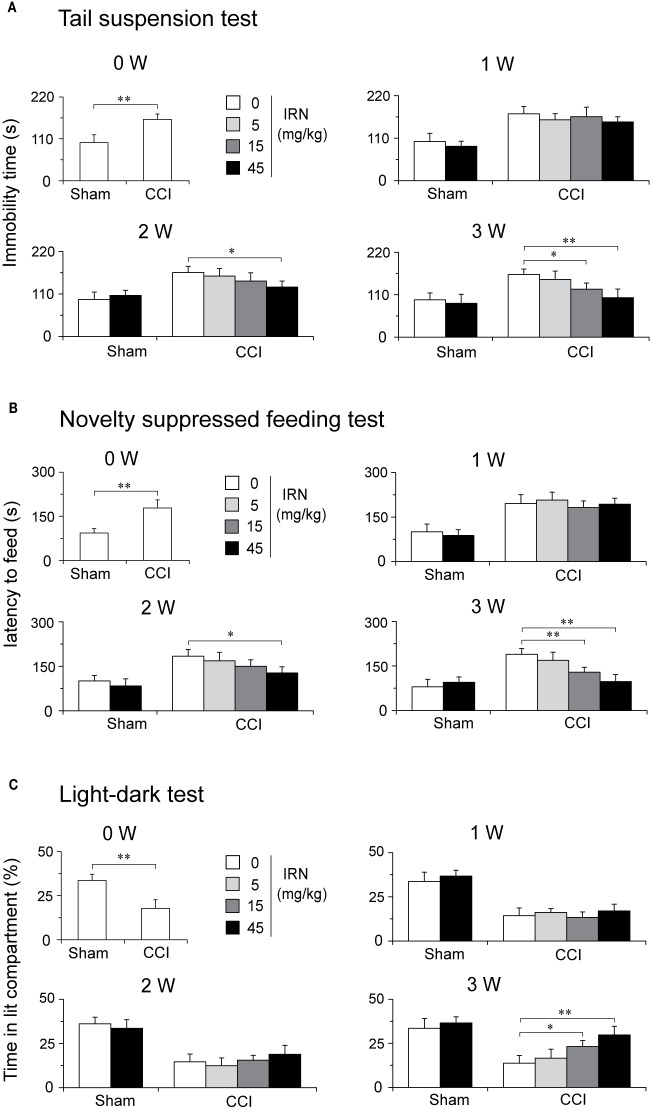
Treatment of neuropathic mice with isorhynchophylline (IRN) ameliorated the co-morbidly behavioral symptoms of depression and anxiety. **(A)** In the tail suspension test (TST), repetitive treatment with isorhynchophylline (5, 15, and 45 mg/kg, p.o., twice per day for 3 weeks) time- and dose-dependently decreased the immobility time in neuropathic mice, without influencing the measures in sham-operated mice. **(B)** In the novelty suppressed feeding test (NSFT), repetitive treatment with isorhynchophylline (5, 15, and 45 mg/kg, p.o., twice per day for 3 weeks) time- and dose-dependently decreased the latency to feed in neuropathic mice, without influencing the measures in sham-operated mice. **(C)** In the light-dark test (LDT), repetitive treatment with isorhynchophylline (5, 15, and 45 mg/kg, p.o., twice per day for 3 weeks) time- and dose-dependently attenuated the anxiety-like behavior (shown as decreased time in lit compartment) in neuropathic mice, without influencing the measures in sham-operated mice. Data are expressed as mean ± SEM (n = 9–12 per group), assessed by one-way ANOVA followed by Student-Newman-Keuls test. *p < 0.05 and **p < 0.01, significantly different from the vehicle group.

After 3 weeks of treatment, isorhynchophylline attenuated in CCI mice these co-morbidly behavioral phenotypes of depression and anxiety in a dose-dependent manner (5, 15, and 45 mg/kg). (a) In the TST, isorhynchophylline dose-dependently decreased the immobility time (F_3,38_ = 13.0, *p* = 0.0127; **Figure 9A**); (b) In the NSFT, isorhynchophylline dose-dependently reduced the latency to feeding (F_3,38_ = 6.2, *p* = 0.0071; **Figure 9B**); (c) In the LDT, isorhynchophylline dose-dependently increased the staying time in lit part (F_3,38_ = 11.8, *p* = 0.0089; **Figure 9C**). These results reveal that isorhynchophylline displayed disease-modifying action up the co-morbidly depressive/anxiogenic-like behaviors specifically in neuropathic mice. Nonetheless, chronic isorhynchophylline treatment did not influence the measures of depressive/anxiogenic-like behaviors in sham-operated mice.

## Discussion

The main finding of this study is the antinociceptive efficacy of isorhynchophylline upon thermal (heat) hyperalgesia and tactile allodynia in neuropathic mice following 2 weeks of oral treatment. To the best knowledge of ours, it is the first report to demonstrate the antinociceptive property of isorhynchophylline in the setting of chronic neuropathic pain, thus may open a new avenue to probe its pharmacological activity in pain-related animal models.

The present antinociceptive activity of isorhynchophylline may incorporate several characteristics. First, isorhynchophylline produced a disease-modifying action, since the dosing started 15 days following peripheral nerve injury when the hypersensitivity had been developed. The reversing action indicates its usefulness in the development of novel painkiller agents. This is important because actually no mechanistically novel painkillers were translated into clinical utility in the past 50 years (Kissin, 2010). Accordingly, it is interesting to know whether isorhynchophylline is able to exert prophylactic action on pain-related behaviors by preemptive dosing. Second, its antinociceptive actions is a genuine one since the pain-related behaviors were assayed on the next morning following the last isorhynchophylline administration and its action was present for at least 1 day. Furthermore, when the consecutive isorhynchophylline administration was interrupted, we observed its antinociception was able to last at least 3 days. These outcomes suggest isorhynchophylline is able to produce persistent antinociception in the context of chronic neuropathic pain. Third, when reinitiated ten days following interruption, isorhynchophylline produced relatively fast onset of antinociception compared with that of the first regimen, implying a process of neural plasticity being involved following repetitive isorhynchophylline treatment.

The descending monoaminergic projection is a vital pathway of endogenous pain modulation (Millan, 2002). Because the escalated monoaminergic tone was reported to correlate with the antidepressant-like effect of isorhynchophylline, we reasoned isorhynchophylline may modulate descending monoaminergic system and thereby exerting its antinociceptive effects. This assumption was supported by the results of escalated spinal monoamine especially 5-HT levels, as well as suppressed 5-HIAA/5-HT ratio and MAO-A activity in neuropathic mice, following repetitive isorhynchophylline treatment. Although these results reveal relevance of spinal monoamine level and isorhynchophylline analgesia, a question however arises as whether the escalated monoamine tone is causally responsible for the disease-modifying action of isorhynchophylline, or it was only an outcome of pain relief. To address this question, we used pharmacological manipulations to ablate spinal NA or 5-HT in mice and examined whether the present isorhynchophylline antinociception was influenced by dearth of monoamine. The results showed pharmacological ablation of spinal 5-HT rather than NA abrogated the antinociceptive effects of isorhynchophylline on thermal hyperalgesia and tactile allodynia, indicating an exceptional serotonergic mechanism being involved. This assertion is supported by another line of evidence showing that pretreatment with 5-HTP (a precursor for 5-HT synthesis) strengthened the isorhynchophylline antinociception.

To further explore the receptor mechanisms underlying isorhynchophylline antinociception, we used several subtype-preferring 5-HT antagonists with high selectivity (affinity) and potency. For example, the binding of WAY-100635 to 5-HT_1A_ receptors is more than 100-fold selective compared to other 5-HT receptor subtypes and major neurotransmitter receptors, reuptake, and ion channel sites (Forster et al., 1995); Isamoltane showed 27-fold higher selectivity at 5-HT_1B_ sites as compared to the binding to 5-HT_1A_ receptors (Waldmeier et al., 1988); Ritanserin showed high affinity binding to 5-HT_2_ receptors, with more than 1,000-fold selectivity compared to 5-HT_1_, and 39-, 77-, 107-, and 166-fold selectivity compared to histamine-H_1_, dopamine-D_2_, and adrenergic-α1 andα2, respectively (Leysen et al., 1985); Ondansetron (GR 38032) possessed a selectivity ratio of more than 1,000-fold for 5-HT_3_ over 5-HT_1_ and 5-HT_2_ sites (Butler et al., 1988). Using these antagonists, we showed the present antinociceptive effects of isorhynchophylline were abolished by WAY-100635, but it was not the case for other antagonists (isamoltane, ritanserin and ondansetron). These results unequivocally reflect the vital role of 5-HT_1A_ receptors in mediating the present antinociceptive effects of isorhynchophylline. Furthermore, the responding 5-HT_1A_ receptors were localized at spinal level, since i.t. but not i.c.v. or i.pl. injection of WAY-100635 counteracted the present isorhynchophylline analgesia. Together, it is feasible to ascribe the antinociceptive mechanism of isorhynchophylline to the spinal 5-HT_1A_ receptors, upon activation by escalated spinal 5-HT tone following repetitive isorhynchophylline treatment.

In another set of *in vitro* tests, we show that isorhynchophylline, at the concentrations ranging from 0.1 to 10 nM, can enhance the activation of 5-HT_1A_ receptor by 8-OH-DPAT in CHO cells, although it seems not to show binding activity, at the concentration range of 0.1 nM–10 μM (up to 1000-fold of isorhynchophylline concentration that enhanced 8-OH-DPAT-induced stimulation of [^35^S] GTPγS binding to CHO cell membranes), upon 5-HT_1A_ receptor in the same membranes. In addition, isorhynchophylline at the concentration range of 0.1–10 nM (the concentrations at which isorhynchophylline can enhance the activation of 5-HT_1A_ receptor by 8-OH-DPAT) did not influence the binding of 8-OH-DPAT to 5-HT_1A_ receptors. These outcomes suggest that isorhynchophylline can modulate the activation of 5-HT_1A_ indirectly, rather than by binding to their orthosteric sites directly. As 5-HT_1A_ is located in both pre- and post-synaptic sites, it is intriguing to determine in future studies the precise synaptic site for isorhynchophylline action, whether post-synaptic pathway mediated by tonic serotonergic transmission or pre-synaptic pathway mediated by receptor desensitization.

Co-morbid mood disorders, such as depression and anxiety, are frequently observed in individuals who suffer neuropathic pain (Gustorff et al., 2008; Naranjo et al., 2019), but relative sparse reports examined the effects of analgesics on co-morbidly affective disabilities. In this study, we also evaluated the effect of repetitive isorhynchophylline treatment on co-morbid symptoms of depression and anxiety in neuropathic mice. We used tail suspension test rather than forced swim test to assay depressive-like behavior, because the CCI induced sectioning of motor fibers may impair the swimming movements (unpublished observation) and hence, a matter of economy. The other behavioral tests are based on behavioral conflict, with light-dark test revealing anxiety and novelty suppressed feeding test revealing both anxiety and depression. Following 3 weeks of treatment, isorhynchophylline ameliorated dose-dependently the behavioral symptoms of depression and anxiety in neuropathic mice. The delayed onset of actions in neuropathic mice and paucity of action in sham mice may reflect altered neural plasticity by chronic isorhynchophylline treatment, and whether the amelioration of co-morbid mood disability has relevance to the relief of concurrent pain symptom is an interesting question. Although the present study did not uncover the precise mechanisms regarding the ameliorative effects of isorhynchophylline on co-morbid mood disability in the context of neuropathic pain, the beneficial actions observed may favor its translational utility in combating persistent neuropathic pain.

Taken together, the present study provides the first line of evidence showing that the natural flavonoid isorhynchophylline, when orally and repetitively administered to mice with neuropathic pain, exerted antinociceptive effects on thermal (heat) hyperalgesia and tactile allodynia. Mechanistically, the antinociception of isorhynchophylline on neuropathic hypersensitivity may depend on the escalated spinal 5-HT tone, which in turn activates spinal 5-HT_1A_ receptors. Over time, neuropathic mice developed behavioral symptoms of mood disability such as anxiety and depression. Our findings also reveal that repetitive isorhynchophylline treatment is able to ameliorate these co-morbid phenotypes, and thereby strengthening its translational value for the treatment of neuropathic pain.

## Data Availability Statement

All datasets generated for this study are included in the article/**Supplementary Material**.

## Ethics Statement

The animal study was reviewed and approved by the Ningbo University Committee on Animal Care and Use.

## Author Contributions

All the authors contributed to the design of the experiments. K-XG, QZ, G-RW, LY, and J-YW performed the experiments. K-XG and XZ wrote and revised the manuscript. QZ and XZ jointly supervised the study.

## Funding

This project was sponsored by the National Natural Science Foundation of China (81541087), the Innovative Research Team of Ningbo (2015C110026), K. C. Wong Magna Fund in Ningbo University, the Construction Project of Special Disease with Clinical Characteristics of Health System in Shanghai Putuo District (2019, Stroke), and the Personnel Training of Overseas Chinese Medicine Center (A1-U17205010502).

## Conflict of Interest

The authors declare that the research was conducted in the absence of any commercial or financial relationships that could be construed as a potential conflict of interest.

## Supplementary Material

Figure S1 Effects of acute isorhynchophylline (IRN) administration on thermal (heat) latency and tactile threshold in sham-operated and neuropathic mice. Acute isorhynchophylline administration was performed on day-15. Following baseline (0 h) evaluation of heat **(A)** and tactile **(B)** sensitivity, mice were administered with isorhynchophylline (5, 15 and 45 mg/kg) and assayed at 0.5, 1, 2, 3 and 4 hour later. **(A)** There is no alteration in thermal (heat) sensitivity in sham-operated and neuropathic mice after acute administration of isorhynchophylline (5, 15 and 45 mg/kg). **(B)** Acute isorhynchophylline treatment (5, 15 and 45 mg/kg) did not impact on the tactile sensitivity in sham-operated and neuropathic mice. Data are expressed as mean ± SEM (n = 8-10 per group), assessed by multifactor ANOVA followed by Duncan test or one-way ANOVA followed by Student-Newman-Keuls test.

Figure S2 Effects of acute isorhynchophylline (IRN) administration on locomotor activity and motor performance in sham-operated and neuropathic mice. Repetitive isorhynchophylline administration (5, 15 and 45 mg/kg) began on day 15 and behavioral tests (rota-rod test and locomotor test) were performed 2 h before 1^st^ isorhynchophylline administration in the morning. **(A)** Repetitive isorhynchophylline administration did not affect the locomotor activity in sham-operated and neuropathic mice. **(B)** Repetitive isorhynchophylline administration did not affect the motor performance in sham-operated and neuropathic mice. Data are expressed as mean ± SEM (n = 8-10 per group), assessed by multifactor ANOVA followed by Duncan test or one-way ANOVA followed by Student-Newman-Keuls test.

Figure S3 Effect of ablating spinal NA on the antinociceptive effects of isorhynchophylline (IRN) in the Hargreaves test and von Frey test. **(A)** Ablating spinal NA by 6-OHDA (20 μg per mouse) did not affect the antihyperalgesic effect of isorhynchophylline in the Hargreaves test. **(B)** Ablating spinal NA by 6-OHDA (20 μg per mouse) did not affect the antiallodynic effect of isorhynchophylline in the von Frey test. Data are expressed as mean ± SEM (n = 8-10 per group), assessed by multifactor ANOVA followed by Duncan test.

Figure S4 Isorhynchophylline (IRN) did not displace or influence the binding of 8-OH-DPAT to 5-HT_1A_ expressing human CHO cell membranes. **(A)** Displacement of [^3^H]-8-OH-DPAT from specific binding sites in 5-HT_1A_ expressing human CHO cell membranes by 8-OH-DPAT (n = 10) and IRN (n = 10). **(B)** Displacement of [^3^H]-8-OH-DPAT from specific binding sites in 5-HT_1A_ expressing human CHO cell membranes by 8-OH-DPAT in the presence of vehicle (n = 10) or 0.1 nM IRN (n = 10). **(C)** Displacement of [^3^H]-8-OH-DPAT from specific binding sites in 5-HT_1A_ expressing human CHO cell membranes by 8-OH-DPAT in the presence of vehicle (n = 9) or 1 nM IRN (n = 10). **(D)** Displacement of [^3^H]-8-OH-DPAT from specific binding sites in 5-HT_1A_ expressing human CHO cell membranes by 8-OH-DPAT in the presence of vehicle (n = 9) or 10 nM IRN (n = 9). Symbols represent mean values ± SEM.
