# Supplementary figures and images for: Isorhynchophylline Exerts Antinociceptive Effects on Behavioral Hyperalgesia and Allodynia in a Mouse Model of Neuropathic Pain: Evidence of a 5-HT1A Receptor-Mediated Mechanism
Source: Front Pharmacol. 2020 Mar 18;11:318. doi: 10.3389/fphar.2020.00318 (PMC7093567; doi:10.3389/fphar.2020.00318)

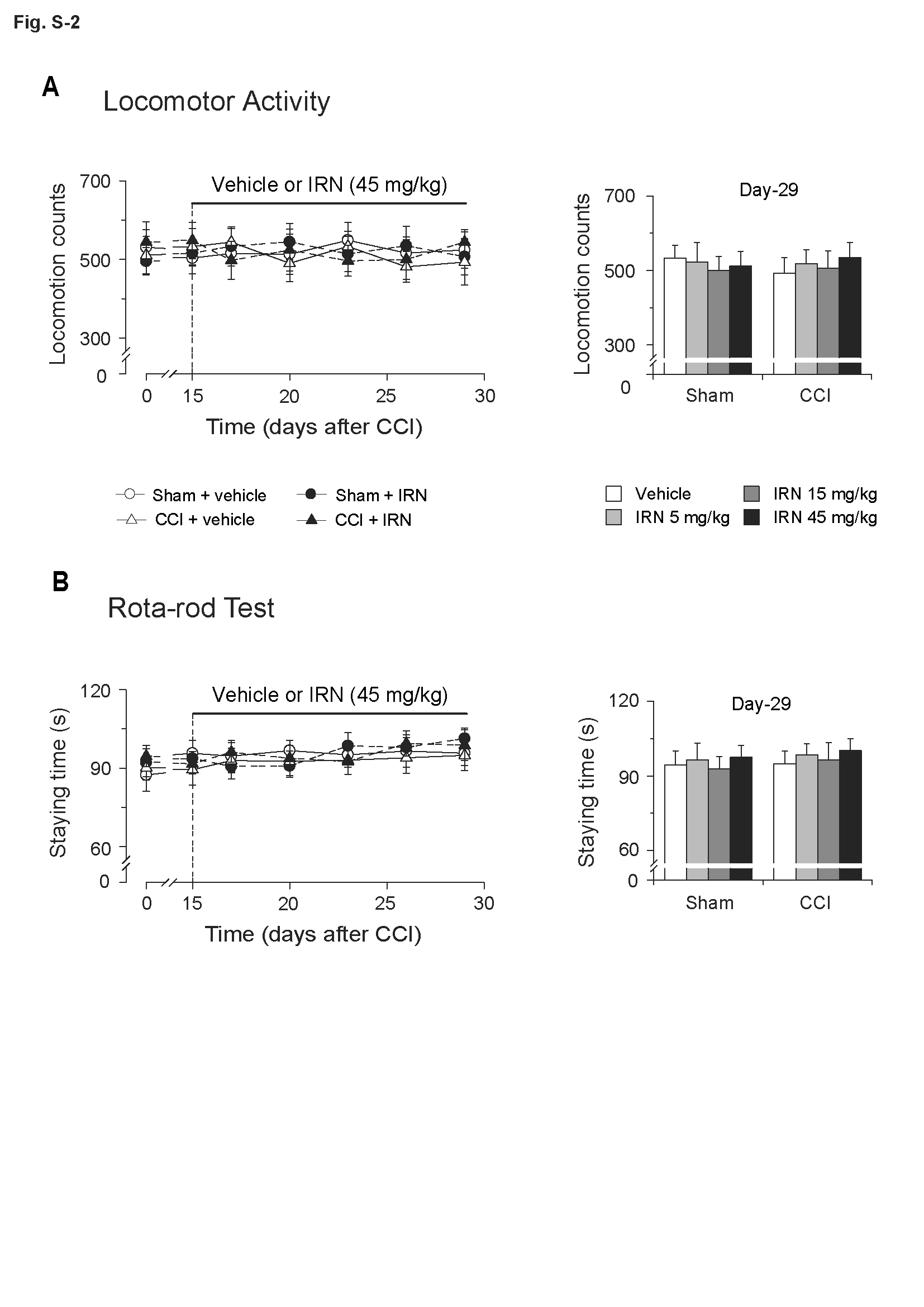

Supplement: Figure S1 — Effects of acute isorhynchophylline (IRN) administration on thermal (heat) latency and tactile threshold in sham-operated and neuropathic mice. Acute isorhynchophylline administration was performed on day-15. Following baseline (0 h) evaluation of heat (A) and tactile (B) sensitivity, mice were administered with isorhynchophylline (5, 15 and 45 mg/kg) and assayed at 0.5, 1, 2, 3 and 4 hour later. (A) There is no alteration in thermal (heat) sensitivity in sham-operated and neuropathic mice after acute administration of isorhynchophylline (5, 15 and 45 mg/kg). (B) Acute isorhynchophylline treatment (5, 15 and 45 mg/kg) did not impact on the tactile sensitivity in sham-operated and neuropathic mice. Data are expressed as mean ± SEM (n = 8-10 per group), assessed by multifactor ANOVA followed by Duncan test or one-way ANOVA followed by Student-Newman-Keuls test. [file Image_1.tif]

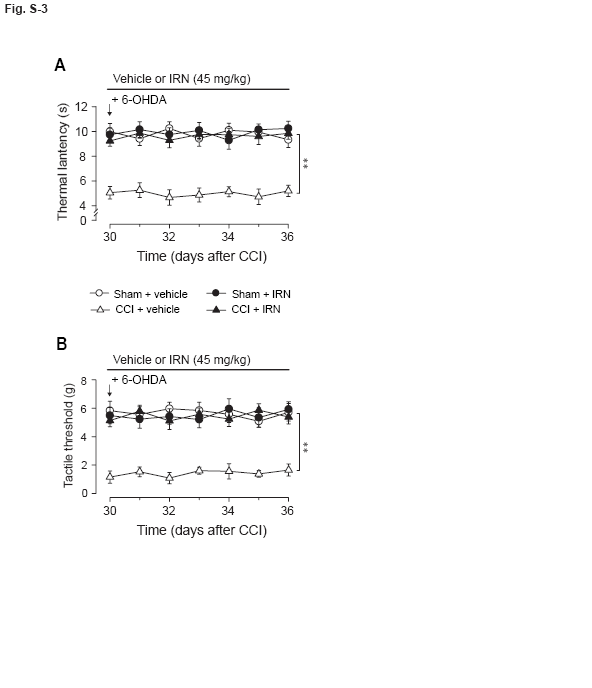

Supplement: Figure S2 — Effects of acute isorhynchophylline (IRN) administration on locomotor activity and motor performance in sham-operated and neuropathic mice. Repetitive isorhynchophylline administration (5, 15 and 45 mg/kg) began on day 15 and behavioral tests (rota-rod test and locomotor test) were performed 2 h before 1st isorhynchophylline administration in the morning. (A) Repetitive isorhynchophylline administration did not affect the locomotor activity in sham-operated and neuropathic mice. (B) Repetitive isorhynchophylline administration did not affect the motor performance in sham-operated and neuropathic mice. Data are expressed as mean ± SEM (n = 8-10 per group), assessed by multifactor ANOVA followed by Duncan test or one-way ANOVA followed by Student-Newman-Keuls test. [file Image_2.tif]

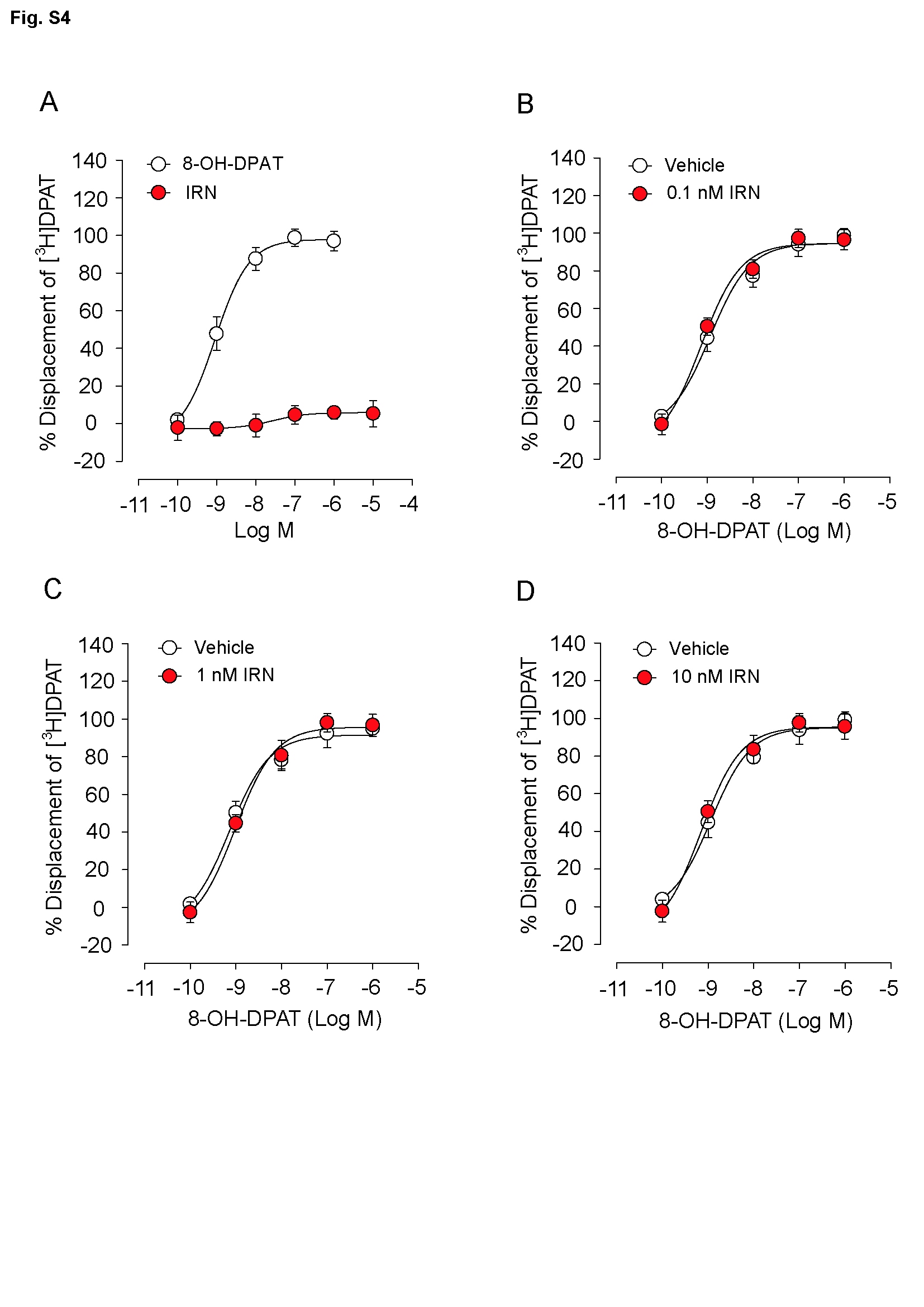

Supplement: Figure S3 — Effect of ablating spinal NA on the antinociceptive effects of isorhynchophylline (IRN) in the Hargreaves test and von Frey test. (A) Ablating spinal NA by 6-OHDA (20 μg per mouse) did not affect the antihyperalgesic effect of isorhynchophylline in the Hargreaves test. (B) Ablating spinal NA by 6-OHDA (20 μg per mouse) did not affect the antiallodynic effect of isorhynchophylline in the von Frey test. Data are expressed as mean ± SEM (n = 8-10 per group), assessed by multifactor ANOVA followed by Duncan test. [file Image_3.tif]

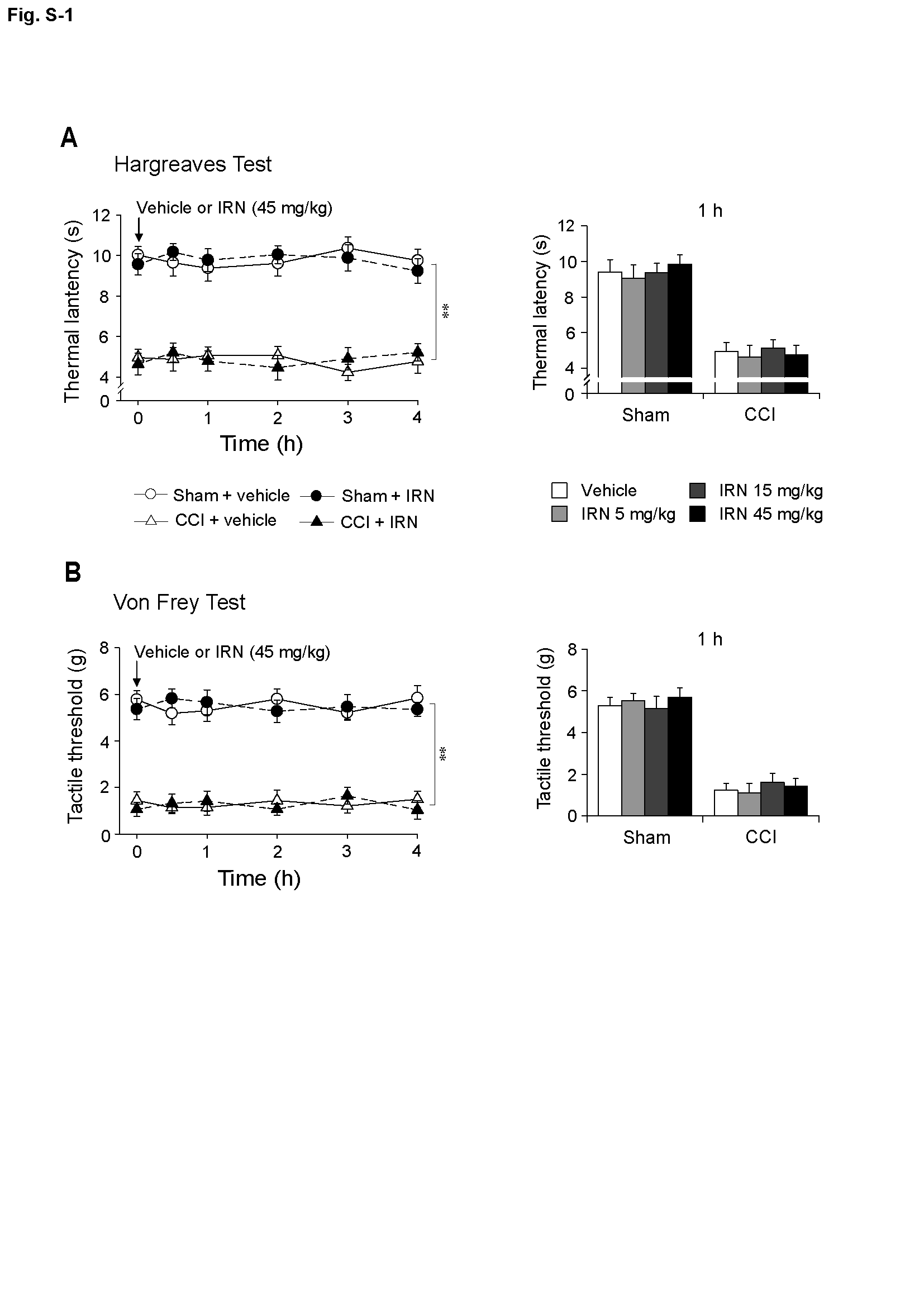

Supplement: Figure S4 — Isorhynchophylline (IRN) did not displace or influence the binding of 8-OH-DPAT to 5-HT1A expressing human CHO cell membranes. (A) Displacement of [3H]-8-OH-DPAT from specific binding sites in 5-HT1A expressing human CHO cell membranes by 8-OH-DPAT (n = 10) and IRN (n = 10). (B) Displacement of [3H]-8-OH-DPAT from specific binding sites in 5-HT1A expressing human CHO cell membranes by 8-OH-DPAT in the presence of vehicle (n = 10) or 0.1 nM IRN (n = 10). (C) Displacement of [3H]-8-OH-DPAT from specific binding sites in 5-HT1A expressing human CHO cell membranes by 8-OH-DPAT in the presence of vehicle (n = 9) or 1 nM IRN (n = 10). (D) Displacement of [3H]-8-OH-DPAT from specific binding sites in 5-HT1A expressing human CHO cell membranes by 8-OH-DPAT in the presence of vehicle (n = 9) or 10 nM IRN (n = 9). Symbols represent mean values ± SEM. [file Image_4.tif]
